# Naïve-pooled pharmacokinetic analysis of pyrazinamide, isoniazid and rifampicin in plasma and cerebrospinal fluid of Vietnamese children with tuberculous meningitis

**DOI:** 10.1186/s12879-016-1470-x

**Published:** 2016-04-02

**Authors:** Thomas Pouplin, Nguyen Duc Bang, Pham Van Toi, Pham Nguyen Phuong, Nguyen Huy Dung, Tran Ngoc Duong, Maxine Caws, Guy E. Thwaites, Joel Tarning, Jeremy N. Day

**Affiliations:** Mahidol-Oxford Tropical Medicine Research Unit, Faculty of Tropical Medicine, Mahidol University, Bangkok, Thailand; Centre for Tropical Medicine and Global Health, Nuffield Department of Medicine, University of Oxford, Oxford, UK; Oxford University Clinical Research Unit, Wellcome Trust Major Overseas Programme, Ho Chi Minh City, Viet Nam; Pham Ngoc Thach Hospital, Ho Chi Minh City, Viet Nam; Liverpool School of Tropical Medicine, Liverpool, UK

**Keywords:** TB, Meningitis, Children, Pharmacokinetics, Rifampicin, Anti-tuberculosis drugs, HPLC

## Abstract

**Background:**

Among the various forms of TB, tuberculous meningitis (TBM) is the most severe, with about 30 % mortality and 50 % of survivors left with neurological sequelae. Children suffer more frequently from TBM than adults and outcomes are often poor due to difficulties in making the diagnosis and uncertainty regarding the best anti-tuberculosis drug regimen. The aim of this prospective study was to describe the pharmacokinetics of pyrazinamide, isoniazid and rifampicin in plasma and cerebrospinal fluid of children with tuberculous meningitis treated with the standard TBM regimen.

**Methods:**

We performed a prospective observational study of 100 consecutively treated children (≤15 years of age) with tuberculous meningitis in Ho Chi Minh City, Vietnam. Children were treated according to the 2006 WHO recommended pediatric treatment regimen consisting of isoniazid (5 mg/kg), rifampicin (10 mg/kg) and ethambutol (15 mg/kg) for 8 months, with the addition of pyrazinamide (25 mg/kg) for the first 3 months and streptomycin (15 mg/kg) for the first 2 months. Pyrazinamide, isoniazid and rifampicin concentrations were measured in plasma at day 14 and in cerebrospinal fluid (CSF) at 1 month by HPLC-UV. A naïve-pooled non-compartmental data analysis was used to describe the pharmacokinetic properties of drugs in the two-age groups of children ≤ 4 years or > 4 years of age.

**Results:**

Younger children, when compared to older children, presented a higher body weight-normalized clearance and volume of distribution, and lower median total plasma exposures for the three studied drugs with −14 %, −22 % and −16 % for Pyrazinamide, Isoniazid and Rifampicin, respectively. In CSF, individual concentrations of isoniazid and pyrazinamide were comparable to that in plasma in both age groups; but rifampicin concentrations were lower than the minimum inhibitory concentration of susceptible bacteria in all but two children.

**Conclusions:**

There is an age-dependent variation in the plasma and cerebrospinal fluid pharmacokinetics of rifampicin, isoniazid and pyrazinamide. The safety and efficacy of higher doses of rifampicin should be investigated for the treatment of childhood tuberculous meningitis.

## Background

Tuberculosis (TB) remains one of the major diseases afflicting children. Among the various forms of TB, tuberculous meningitis (TBM) is the most severe, with about 30 % mortality and 50 % of survivors left with neurological sequelae [1, 2]. Children suffer more frequently from TBM than adults and outcomes are often poor due to difficulties in making the diagnosis, which can delay treatment, and uncertainty regarding the best anti-tuberculosis drug regimen. More than 60 years after the development of the first anti-TB drugs, the optimal treatment of childhood TBM is still uncertain and clinical care is almost exclusively extrapolated from studies in adults. The different multiple drug regimens used in TBM are not uniform and mostly extrapolated from those used in pulmonary TB, with very little supportive evidence.

The first line anti-TB drugs are known to have differing cerebrospinal fluid (CSF) penetration. While plasma and CSF exposure tend to be similar for isoniazid and pyrazinamide, the penetration of rifampicin in CSF is poor, as it is for ethambutol [3–6]. The death rate of TBM patients may reflect both the poor penetration of drugs into the brain and the poor antibacterial activity at the current dosages. Whilst the pharmacokinetics of first-line TB drugs has been extensively studied in plasma in adults, only few published data describe exposure of these drugs in plasma and CSF of children. Higher doses and maybe different agents to those used for pulmonary TB may be required to improve outcomes from childhood TBM, but there are scant data to determine the best therapeutic approach. New randomized controlled trials have emerged from the literature, trying to assess experimental regimens in TBM [7–9]. However, evaluating modifications in a multiple drug regimen is complex and complicated trial designs can be required to measure and separate the effect of combination treatment [10].

Our aim was to perform a naïve-pooled data non-compartmental pharmacokinetic analysis of pyrazinamide, isoniazid and rifampicin in plasma and CSF in the treatment of childhood TBM and to determine whether age influenced drug exposure.

## Methods

### Patients

The study was a prospective observational study of 100 patients under 15 years of age presenting with suspected TBM to the Pham Ngoc Thach Hospital for Tuberculosis and Lung Diseases (PNT) in Ho Chi Minh City. This hospital is the tertiary referral centre for patients with TB from the whole of southern Vietnam (population around 45 million). All patients aged ≤15 years presenting to PNT with meningitis symptoms and signs (e.g. fever, headache, neck stiffness, vomiting, confusion, coma, convulsions, cranial nerve palsies, hemiplegia or paraplegia) and who were suspected to have TBM by their attending physician were eligible to enter the study. Patients were graded for severity at the point of study entry. For children more than 5 years of age severity was graded using the modified UK Medical Research Council (MRC) criteria, based on Glasgow Coma Score (GCS). For children less than 5 years of age, severity was graded according to the Blantyre Coma Score (BCS).

Ethical approval for the study was granted from the ethical review board of Pham Ngoc Thach Hospital, Viet Nam, Health Services of Ho Chi Minh City and the Oxford Tropical Research Ethics Committee (OxTREC), UK. Written informed consent was obtained from the parents or guardians of all participants.

### Anti-tuberculosis drug treatment

Children diagnosed with TBM were treated with the standard Vietnamese National TB Programme (NTP) 8-month regimen. At the time the study was initiated, Vietnam had not yet adopted the new doses recommended by WHO [11] in 2011. Therefore, children received once daily treatment with pyrazinamide (25 mg/kg), isoniazid (5 mg/kg), rifampicin (10 mg/kg), ethambutol (15 mg/kg) and streptomycin (15 mg/kg) for the first 2 months; then isoniazid, rifampicin, ethambutol and pyrazinamide for the third month; and then isoniazid, rifampicin and ethambutol for the continuation phase of 5 months. Dexamethasone was used as adjunctive therapy for the first 6 (MRC/BCS grade I disease) or 8 (MRC/BCS grades II or III disease) weeks of treatment in all patients according to standard practice [12].

Locally produced adult fixed dose combination (FDC) tablets were used for treatment (Turbezid® Namha Pharmaceutical Joint-Stock Company). These consisted of film-coated and scored tablets containing 150 mg of rifampicin, 75 mg of isoniazid and 400 mg of pyrazinamide. Each tablet was designed for ≥ 15 kg of body weight and used a drug ratio compatible with the adult TB regimen. Tablets were split in thirds for the lightest patients (<5 kg), halves (5–7.5 kg), 2 thirds (7.5–10 kg) or used as whole plus extra split for over 15 kg. Ethambutol was given orally as a single drug tablet and streptomycin was injected intravenously.

### Pharmacokinetic sampling

Each patient enrolled in the study was asked to provide plasma on day 1 (first day of treatment), day 14 (steady-state), and plasma and CSF at months 1 and 3. Enrolled patients were stratified into two groups (below and above 4 years of age) and randomisation of plasma sampling times was stratified by group. Previous admission patterns led us to believe that this age grouping would deliver to groups of approximately equal size. For each patient, two plasma samples were collected at two of ten randomly allocated pre-specified time points (i.e. at 1, 2, 3, 4, 5, 6, 8, 12, 18 or 24 h post-dose) on each of days 1 and 14. One additional plasma sample was collected for each patient at month 1 and 3, again at a randomly allocated time points between 3 to 5 h post-dose. Each child provided a maximum of 6 plasma samples over the trial.

CSF sampling by lumbar puncture (LP) was conformed to standard clinical care with CSF examination executed to assess treatment response. LPs were performed at months 1 and 3 post study entry at randomly allocated time points either 3, 4 or 5 h post-dose. Each LP was followed by a blood draw performed within 15 min. Freshly collected clinical samples were immediately processed and stored at −80 °C pending bioanalysis.

### Acetylator genotyping

Acetylator genotype and predicted phenotype for each patient was determined by sequencing the second exon of the arylamine N-acetyltransferase 2 (NAT2) gene which contains the functional polymorphisms [13, 14]. Genomic DNA was extracted from blood using the Nucleon Genomic DNA extraction kit (GE Healthcare, Amersham, United Kingdom). Two primers NAT2F 5′-TGGGCTTAGAGGCTATTT and NAT2R 5′-GAGTTGGGTGATACATACAC were designed using Primer Express version 2.0 software (Applied Biosystems Inc, Foster City, CA, USA) to amplify a 768 bp sequence of the second exon of the NAT2 gene which contains the relevant polymorphisms (G191A, C282T, T341C, C481T, G590A, A803G, G857A). Following purification (QIAgen PCR purification kits, QIAGEN, United Kingdom) amplicons were sequenced (3130xl Genetic Analyzer Applied Biosystem, Hitachi, Singapore) and phenotypes predicted from genotypes previously defined in a cohort of healthy Vietnamese volunteers in a study correlating urinary caffeine metabolites ratios with NAT2 genotypes (data not shown) [15, 16].

### Drug bioanalysis

The concentrations of pyrazinamide, isoniazid and rifampicin in plasma and CSF were measured by a fully validated HPLC method with UV detection following the Food and Drug Administration (FDA) and International Conference on Harmonization (ICH) recommendations [17]. Ethambutol is only detectable with mass spectrometry and was not analysed in this study. Thawed samples (plasma or CSF) were mixed for at least 30 s and left undisturbed on the bench for further 10 min. Then, 300 μL of IS solution (Guanosine and Phenacetine in saturated NaCl) were added to 300 μL of sample (plasma or CSF) in a 1.5 mL microcentrifuge tube. The tube was mixed for 15 s and left at room temperature for 10 min followed by a centrifugation at 3000 g for 5 min. The samples were loaded into a conditioned ISOLUTE-C18 50 mg/mL 96 well-plate (Biotage, Uppsala, Sweden) and washed with 0.5 mL of saturated NaCl until dryness. Isoniazid, pyrazinamide and guanosine were eluted with 2 × 250 μL of methanol–water (10:90, v/v). A second elution containing rifampicin and phenacetine was carried out by using 250 μL acetonitrile followed by 250 μL methanol. Ten μL of each eluate were injected into 2 independent equilibrated LC-UV systems (Lachrom Elite – Hitachi – Merck system). Separation was performed on a 5 μm LichroCart 125 × 4.6 mm Purosphere RP-18 end-capped column, equipped with 5 μm guard column LichroCart 4 × 4 mm, RP-18e (Merck, Darmstadt, Germany), and an isocratic flow rate of 1 mL/min of phosphate buffer (pH 4.2, 50 mM) and acetonitrile 0.25 % (v/v) for isoniazid, pyrazinamide and guanosine, and phosphate buffer (pH 4.2, 50 mM) and acetonitrile 36 % (v/v) for rifampicin and phenacetine. The chromatography time for all assays was set to 7 min with detection at 263 nm for isoniazid, pyrazinamide and guanosine and 247 nm for rifampicin and phenacetine, respectively. In plasma, the calibration ranged [lower limit of quantification (LOQ) to upper limit of quantification (ULOQ)] from 0.2 to 80 μg/mL, 0.2 to 32 μg/mL and 0.1 to 20 μg/mL for pyrazinamide, isoniazid and rifampicin, respectively. In CSF, the ranges were 0.2 to 40 μg/mL, 0.2 to 32 μg/mL and 0.1 to 16 μg/mL for pyrazinamide, isoniazid and rifampicin, respectively. Triplicates of quality control samples (low, middle and high) were analyzed within each batch to ensure accuracy and precision throughout the analysis. The precision of all quality control samples were below 15 % CV during routine drug measurements.

### Naïve-pooled non-compartmental analysis

The naïve-pooled data analysis was based on plasma and CSF data collected at the first steady-state occasion (i.e. at day 14 for plasma samples and at month 1 for CSF samples). Drugs reach their respective steady-states after a minimum of 5 half-lives. With anti-TB drugs, the steady-state is assumed to be reached after 2 weeks even for RIF and its well described autoinduction metabolism [18, 19]. We also anticipated no significant pharmacokinetic changes between day 14 and month 1. The concentration-time data were pooled dependent on sampling matrix and split into 2 independent age-groups: ≤ 4 years and > 4 years. All concentration-time data were pooled for the following time windows: 0–0.5, 0.5–1, 1–2, 2–3, 3–4, 4–5, 5–6, 6–8, 8–12, 12–18, and 18–24 h post-dose. The 25^th^ percentile (Q1), 50^th^ percentile (median), and 75^th^ percentile (Q3) of drug concentrations were calculated within each time window. Drug measurements below the LOQ were set to LOQ/2 to avoid bias on account of data censoring. Median concentrations and mid-time intervals were used in the non-compartmental analysis, and Q1 and Q3 were used for graphical representation only.

Naïve-pooled concentration-time data were evaluated using a non-compartmental analysis approach implemented in WinNonlin version 6.3 (Pharsight Corporation, California, USA). Median drug exposure up to the last time point (AUC_0-t_) was calculated using the linear trapezoidal method for ascending concentrations and the logarithmic trapezoidal method for descending concentrations. The median terminal elimination rate constant (λ_Z_) was determined as the slope of the terminal elimination phase by best-fit ordinary linear regression. Drug exposure was extrapolated from the last observed median concentration to time infinity by C_LAST_/λ_Z_ for each individual group to compute total drug exposure (AUC_0-∞_). The terminal elimination half-life (t_1/2_) was calculated as ln(2)/λ_Z_. Maximum median concentration (C_MAX_) and time to C_MAX_ (T_MAX_) were taken directly from the observed data. Apparent median volume of distribution (V_Z_/F) and median oral clearance (CL/F) were computed according to standard procedures. The wild-type minimum inhibitory concentrations (MIC) were arbitrary chosen from reference values from the literature [20, 21]. They were set at 12.5 mg/L, 0.2 mg/L and 1.0 mg/L for pyrazinamide, isoniazid and rifampicin, respectively. These MIC values were implemented in WinNonlin for the calculation of time above MIC (T > MIC) and exposure above MIC (AUC > MIC) as therapeutic indices. Sub-therapeutic thresholds were applied using maximum plasma concentrations (C_MAX_) associated with poor outcome in adults with pulmonary TB [18, 22–25]. The use of these cut-off concentrations is difficult in the context of a multiple drugs therapy and they are still controversial and debated in the literature. However, although there are no consensus values for sub-therapeutic thresholds associated with TBM, or in children, they can be considered as “biomarkers” of clinical outcome. More importantly even if only used graphically in this study, they are able to visualize under-exposure and compare peak concentrations between observed values in children and an adult reference. The sub-therapeutic thresholds were set at 35 μg/mL, 3 μg/mL and 8 μg/mL for pyrazinamide, isoniazid and rifampicin, respectively. Naïve-pooled pharmacokinetic parameter estimates are summarized in Tables 2 to 5, and the relative differences (%) in individual median parameter estimates were calculated between the two age strata for each drug, using the older age group as a reference.

## Results

### Clinical study characteristics

One hundred children were enrolled in this study. The overall median age of the studied children was 36 months with the largest proportion of children (64 %) below 4 years of age. Among the 33 children ≥ 5 years of age, 16 (48 %) patients were admitted with stage I TBM according to the MRC criteria, 11 (33 %) with stage II and 6 (18 %) with stage III. Among the remaining 67 children < 5 years of age, 43 (64 %) were admitted with a BCS of 4–5 (BCS I), 12 (18 %) with a BCS of 2–3 (BCS II) and 12 (18 %) with a BCS of 0–1 (BCS III). An important proportion (39 %) of the Vietnamese children in this study were classified as malnourished according to the WHO growth criteria with a body mass index (BMI) Z-score below −2 (negative two) [26]. However, a significant weight gain occurred during the first 3 months of treatment (Table 1). The treatment doses in mg/kg, based on the body weight measured at enrollment, did not show significant differences between the two age-groups (Table 2, 3 and 5 for pyrazinamide, isoniazid and rifampicin, respectively).

**Table 1:** Baseline characteristics and outcomes of studied children

	<4 years old^a^	>4 years old^b^	Total
Number of patients enrolled, n	64	36	100
Male/Female, n (male %)	36/65 (56.3)	20/36 (55.6)	56.0
Age (months)	12 (2–48)	96 (48–180)	36 (2–180)
Body weight (kg)	9.0 (4.0–15.0)	19.0 (13.5–43.0)	10.9 (4.0–43.0)
BMI (kg/m^2^)	15.4 (11.9–22.0)	13.3 (10.6–20.2)	14.3 (10.6–22.0)
Malnourished, n (%)	18/64 (28.1)	20/36 (55.5)	38.0
Body weight gained (kg)			
after 1 month	0.5 (−0.3–2.5)	0.75 (−7.5–6.0)	0.5 (−7.5–6.0)
after 3 months	1.3 (−0.8–5.3)	2.0 (−9.0–12.5)	1.5 (−9.0–12.5)
Fast/Slow acetylator status, n (%)^c^	35/23 (60 %)	29/5 (85 %)	64/28 (70 %)
HIV positive, n (%)^d^	2/61 (3.3 %)	2/35 (5.7 %)	4/96 (4.2 %)
TBM Grade I, n (%)	43/67 (64 %)	16/33 (48 %)	59/100 (59 %)
TBM Grade II, n (%)	12/67 (18 %)	11/33 (33 %)	23/100 (23 %)
TBM Grade III, n (%)	12/67 (18 %)	6/33 (18 %)	18/100 (18 %)
TB diagnosis: Definite, n (%)	3/64 (4.7 %)	3/36 (8.3 %)	6/100 (6.0 %)
TB diagnosis: Probable, n (%)	39/64 (61 %)	27/36 (75 %)	66/100 (66 %)
TB diagnosis: Possible, n (%)	22/64 (34 %)	6/36 (17 %)	28/100 (28 %)
Death, n (%)	10/64 (16 %)	5/36 (14 %)	15/100 (15 %)
Permanent Sequelae, n (%)^e^	19/51 (37 %)	8/30 (27 %)	27/81 (33 %)

All values are reported as median (range) unless otherwise specified. ^a^disease severity grade assessed in children below 5 years old by Blantyre Coma Score; ^b^disease severity grade assessed in children over 5 years old by Glasgow Coma Score; ^c^8 patients did not complete the NAT2 genotype; ^d^4 patients out of 100 did not complete HIV testing; ^e^surviving patients were assessed after treatment completion at month 8 (4 were lost to follow-up)

**Table 2:** Pharmacokinetic non-compartmental results of pyrazinamide in children above and below 4 years of age

Pyrazinamide	<4 years old	>4 years old	% difference
Number of patients enrolled	64	36	
Body Weight (median kg - range)	9.0 (4.0–15.0)	19.0 (13.5–43.0)	
Age (median months - range)	12 (2–48)	96 (48–180)	
Dosage (mean - % CV)	4.70 (12.8)	5.12 (9.18)	*p*-value: 0.0001^a^
C_MAX_ (ug/mL)	30.1	37.4	−19.5
T_MAX_ (hr)	2	3	−33.3
CL/F (L/hr)	1.09	1.88	−42
CL/F/BW (L/hr/kg)	0.12	0.1	20
V/F (L)	7.57	12.2	−37.9
V/F/BW (L/kg)	0.84	0.64	31.3
T_1/2_ (hr)	4.82	4.5	7.11
AUC_0-∞_ (hr × ug/mL)	245	284	−13.7
AUC_0-∞_/dosage (hr × ug/mL/(mg/kg))	10.1	10.8	−6.2
T > MIC (hr)	7.28	8.46	−13.9
T > MIC/24 h (%)	30.3	35.2	−13.9
AUC > MIC (hr × ug/mL)	62.3	95.2	−34.6

Apart for dosage with mean and % CV, all values are reported as median (range) or as median estimates for pharmacokinetic parameters. *C*_*MAX*_ maximum observed plasma concentration after oral administration, *T*_*MAX*_ observed time to reach C_MAX_, *CL/F* oral elimination clearance, *CL/F/BW* elimination clearance corrected by the median body weight, *V/F* apparent volume of distribution, *V/F/BW* apparent volume of distribution corrected by the median body weight, *T*_*1/2*_ terminal elimination half-life, *AUC*_*0-∞*_ predicted area under the plasma concentration-time curve after the last dose from zero time to infinity, *T > MIC* time above the minimum inhibitory concentration, *T > MIC/24 h* percentage of the time above the minimum inhibitory concentration over the 24 h dose interval, *AUC > MIC* observed area under the plasma concentration-time curve above the minimum inhibitory concentration. % difference is calculated with the > 4 years group used as the reference. ^a^Mann–Whitney T-test

**Table 3:** Pharmacokinetic non-compartmental results of isoniazid in children above and below 4 years of age

Isoniazid	<4 years old	>4 years old	% difference
Number of patients enrolled	64	36	
Body Weight (median kg - range)	9.0 (4.0–15.0)	19.0 (13.5–43.0)	
Age (median months - range)	12 (2–48)	96 (48–180)	
Dosage (mean - % CV)	25.0 (12.8)	27.3 (9.17)	*p*-value: 0.0002^a^
C_MAX_ (ug/mL)	4.38	5.11	−14.3
T_MAX_ (hr)	2	3	−33.3
CL/F (L/hr)	2.48	3.88	−36.1
CL/F/BW (L/hr/kg)	0.28	0.2	40
V/F (L)	11.3	14.9	−23.8
V/F/BW (L/kg)	1.26	0.78	61.5
T_1/2_ (hr)	3.17	2.66	19.2
AUC0-∞ (hr × ug/mL)	20.2	25.8	−21.8
AUC_0-∞_/dosage (hr × ug/mL/(mg/kg))	4.48	5.26	−14.8
T > MIC (hr)	14.2	14.5	−1.8
T > MIC/24 h (%)	59.3	60.3	−1.79
AUC_0–24 h_ > MIC (hr × ug/mL)	16.3	22	−25.8

Apart for dosage with mean and % CV, all values are reported as median (range) or as median estimates for pharmacokinetic parameters. *C*_*MAX*_ maximum observed plasma concentration after oral administration, *T*_*MAX*_ observed time to reach C_MAX_, *CL/F* oral elimination clearance, *CL/F/BW* elimination clearance corrected by the median body weight, *V/F* apparent volume of distribution, *V/F/BW* apparent volume of distribution corrected by the median body weight, *T*_*1/2*_ terminal elimination half-life, *AUC*_*0-∞*_ predicted area under the plasma concentration-time curve after the last dose from zero time to infinity, *T > MIC* time above the minimum inhibitory concentration, *T > MIC/24 h* percentage of the time above the minimum inhibitory concentration over the 24 h dose interval, *AUC > MIC* observed area under the plasma concentration-time curve above the minimum inhibitory concentration. % difference is calculated with the > 4 years group used as the reference. ^a^Mann–Whitney T-test

Fifteen patients (15 %) died. Ten of 64 children under 4 (16 %) vs 5 of 36 children ≥ 4 years (14 %). The majority of deaths occurred in patients with advanced grades of disease at presentation (MRC grade II or III, or BCS from 0–3), but a single death occurred in a patient presenting with MRC grade-I TBM with concomitant HIV infection. It must be noticed that a higher proportion of older children had definite or probable TBM (30/36: 83 %) compared to younger children (42/64: 66 %) which might influence the respective mortality and morbidity in each group. Eight deaths occurred within the first 6 days, six between 6 and 45 days and the last death occurred at day 92. There were no significant differences in mortality or morbidity between the two age groups. At the end of the treatment, 6 out of 81 children (7 %) had severe symptoms and/or required help day and night, 21 of 81 children (26 %) had intermediate sequelae but independent life (modified Rankin scale from 2–3 [12]), and 54 out of 81 children (67 %) recovered fully (4 children did not complete the final study visit). In this study, AST and ALT levels were measured repeatedly for all patients for 90 days (pre-dose, 30, 60 and 90 days) or at any time the children developed symptoms. Two out of 99 children (2 %) met the criteria for drug-induced liver toxicity (DILI) but no child died due to DILI in this study.

### Pharmacokinetic analysis

All pharmacokinetic blood draws were close to the pre-specified protocol sampling times (<10 % CV for both age-groups). The absolute difference between the actual sampling time and mean time in the assigned window ranged between −8.2 min to +6.4 min for children below 4 years of age, and between −6.5 min to +0.3 min for children above 4 years of age. The density of individual data points used in each bin was also comparable between the two age groups (after adjusting for the different sample size).

**Fig. 1:**
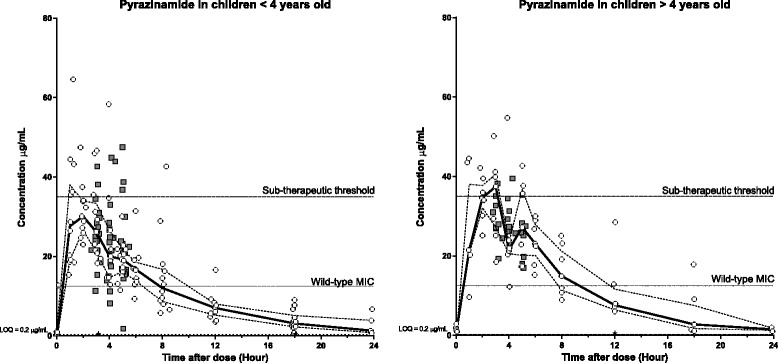
Plasma (open circles) and CSF (grey squares) concentration-time profiles for pyrazinamide stratified for age (<4 years old [left] and > 4 years old [right]). Continuous lines represent the median plasma concentration and broken lines represent the plasma upper and lower quartiles. Black stars represent plasma samples with concentrations below LOQ. Sub-therapeutic threshold of 35 μg/mL; Minimum Inhibitory Concentration (MIC) of 12.5 μg/mL; and Limit of Quantification (LOQ) of 0.2 μg/mL

**Fig. 2:**
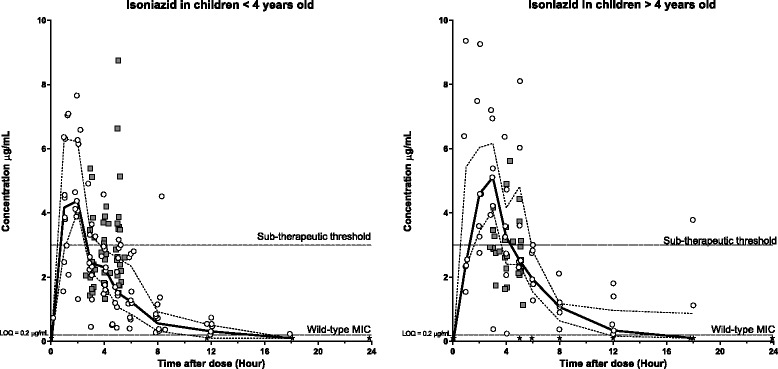
Plasma (open circles) and CSF (grey squares) concentration-time profiles for isoniazid stratified for age (<4 years old [left] and > 4 years old [right]). Continuous lines represent the median plasma concentration and broken lines represent the plasma upper and lower quartiles. Black stars represent plasma samples with concentrations below LOQ. Sub-therapeutic threshold: 3 μg/mL; Minimum Inhibitory Concentration (MIC) and Limit of Quantification (LOQ): 0.2 μg/mL

**Fig. 3:**
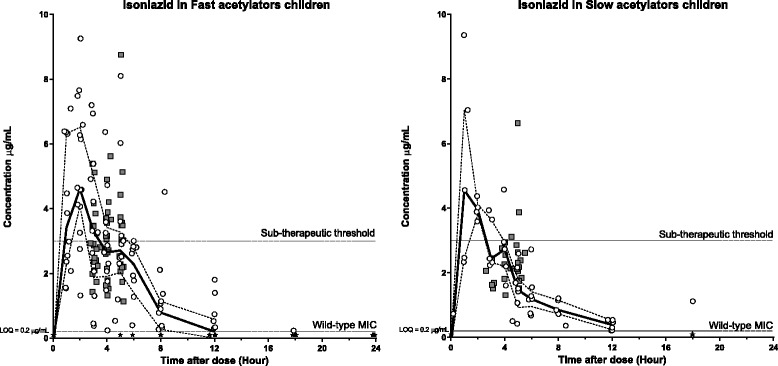
Plasma (open circles) and CSF (grey squares) concentration-time profiles for isoniazid stratified for Fast acetylators [left] and Slow acetylators [right]. Continuous lines represent the median plasma concentration and broken lines represent the plasma upper and lower quartiles. Black stars represent plasma samples with concentrations below LOQ. Sub-therapeutic threshold: 3 μg/mL; Minimum Inhibitory Concentration (MIC) and Limit of Quantification (LOQ): 0.2 μg/mL

**Fig. 4:**
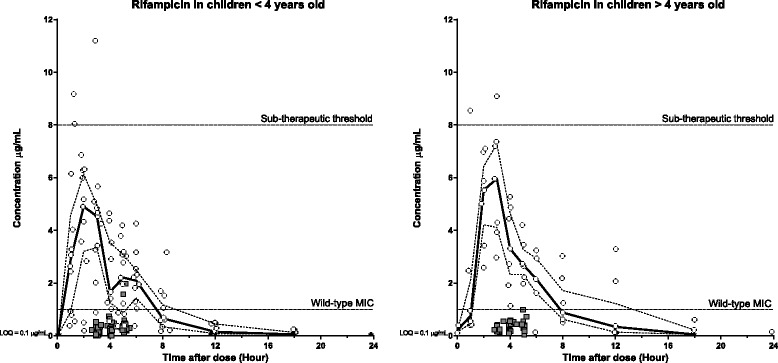
Plasma (open circles) and CSF (grey squares) concentration-time profiles for rifampicin stratified for age (<4 years old [left] and > 4 years old [right]). Continuous lines represent the median plasma concentration and broken lines represent the plasma upper and lower quartiles. Black stars represent plasma samples with concentrations below LOQ. Sub-therapeutic threshold: 8 μg/mL; Minimum Inhibitory Concentration (MIC): 1.0 μg/mL; Limit of Quantification (LOQ): 0.1 μg/mL

The median and inter-quartile range of naïve-pooled plasma concentrations represented the central trends and the variability of the measured concentration-time profiles well in both age-groups for all drugs (Figs. 1, 2, 3 and 4). As seen in Tables 2, 3,4 and 5, the dosages were in agreement with the 2006 WHO recommendations at the time of the study. However, significant differences were found between young and old children for all drugs but INH when the dosing of fast and slow acetylators was compared. This is likely an inevitable consequence of larger relative body weight differences for younger children enhanced by the need to fit the weight bands and the lack of uniformity when tablets have to be split [27].

**Table 4:** Pharmacokinetic non-compartmental results of isoniazid in fast and slow acetylators

Isoniazid	SLOW	FAST	% difference
Number of patients enrolled^a^	28	64	
Body Weight (median kg - range)	10.0 (4.5–26.0)	13.0 (4.4–44.0)	
Age (median months - range)	36 (6–180)	42 (2–180)	
Dosage (mean - % CV)	4.82 (11.7)	4.70 (13.3)	*p*-value: > 0.05^b^
C_MAX_ (ug/mL)	4.56	4.62	−1.3
T_MAX_ (hr)	1	2	−50
CL/F (L/hr)	2.28	2.47	−7.69
CL/F/BW (L/hr/kg)	0.23	0.19	21.1
V/F (L)	12.2	7.05	72.3
V/F/BW (L/kg)	1.21	0.54	124
T_1/2_ (hr)	3.69	1.98	86.4
AUC0-∞ (hr × ug/mL)	21.9	22.8	−3.78
AUC_0-∞_/dosage (hr × ug/mL/(mg/kg))	4.38	4.95	−11.5
T > MIC (hr)	14.8	11.9	24.7
T > MIC/24 h (%)	61.7	49.4	24.8
AUC_0–24 h_ > MIC (hr × ug/mL)	17.7	19.8	−10.7

^a^Acetylator genotype was determined in 92 patients. ^b^Mann–Whitney T-test. Apart for dosage with mean and % CV, all values are reported as median (range) or as median estimates for pharmacokinetic parameters. *C*_*MAX*_ maximum observed plasma concentration after oral administration, *T*_*MAX*_ observed time to reach C_MAX_, *CL/F* oral elimination clearance, *CL/F/BW* elimination clearance corrected by the median body weight, *V/F* apparent volume of distribution, *V/F/BW* apparent volume of distribution corrected by the median body weight, *T*_*1/2*_ terminal elimination half-life, *AUC*_*0-∞*_ predicted area under the plasma concentration-time curve after the last dose from zero time to infinity, *T > MIC* time above the minimum inhibitory concentration, *T > MIC/24 h* percentage of the time above the minimum inhibitory concentration over the 24 h dose interval, *AUC > MIC* observed area under the plasma concentration-time curve above the minimum inhibitory concentration. % difference is calculated with the > 4 years group used as the reference

**Table 5:** Pharmacokinetic non-compartmental results of rifampicin in children above and below 4 years of age

Rifampicin	<4 years old	>4 years old	% difference
Number of patients enrolled	64	36	
Body Weight (median kg - range)	9.0 (4.0–15.0)	19.0 (13.5–43.0)	
Age (median months - range)	12 (2–48)	96 (48–180)	
Dosage (mean - % CV)	9.38 (12.8)	10.2 (9.04)	*p*-value: 0.0002^a^
C_MAX_ (ug/mL)	4.9	5.96	−17.8
T_MAX_ (hr)	2	3	−33.3
CL/F (L/hr)	4.66	7.86	−40.7
CL/F/BW (L/hr/kg)	0.52	0.41	26.8
V/F (L)	15.6	25.5	−38.9
V/F/BW (L/kg)	1.73	1.34	29.1
T_1/2_ (hr)	2.31	2.25	2.67
AUC0-∞ (hr × ug/mL)	21.5	25.4	−15.7
AUC_0-∞_/dosage (hr × ug/mL/(mg/kg))	2.36	2.6	−9.23
T > MIC (hr)	6.89	6.66	3.45
T > MIC/24 h (%)	28.7	27.8	3.39
AUC_0–24 h_ > MIC (hr × ug/mL)	11.7	14.7	−20.1

Apart for dosage with mean and % CV, all values are reported as median (range) or as median estimates for pharmacokinetic parameters. *C*_*MAX*_ maximum observed plasma concentration after oral administration, *T*_*MAX*_ observed time to reach C_MAX_, *CL/F* oral elimination clearance, *CL/F/BW* elimination clearance corrected by the median body weight, *V/F* apparent volume of distribution, *V/F/BW* apparent volume of distribution corrected by the median body weight, *T*_*1/2*_ terminal elimination half-life, *AUC*_*0-∞*_ predicted area under the plasma concentration-time curve after the last dose from zero time to infinity, *T > MIC* time above the minimum inhibitory concentration, *T > MIC/24 h* percentage of the time above the minimum inhibitory concentration over the 24 h dose interval, *AUC > MIC* observed area under the plasma concentration-time curve above the minimum inhibitory concentration. % difference is calculated with the > 4 years group used as the reference. ^a^Mann–Whitney T-test

#### Pyrazinamide

Pooled pharmacokinetic parameters, stratified for children above and below 4 years of age, are presented in Table 2. Young children, as compared to older children, showed a trend of 22 % higher body weight-normalized elimination clearance and 31 % higher volume of distribution, a 20 % lower maximum concentration but a modest decrease of 14 % in total exposure. Total exposure, normalized on dosage (mg/kg), showed a less pronounced decrease with 6.3 %. However, a relatively small decrease of 14 % in the time above MIC was seen while the total exposure above MIC was found lower (35 %) in young children compared to older children. The previously defined sub-therapeutic plasma concentration threshold of 35 μg/mL was only reached by older patients [18, 28]. The distribution and accumulation of pyrazinamide into CSF could only be assessed graphically but showed similar exposure to that in plasma, above the wild-type MIC, in both age groups (Fig. 1).

#### Isoniazid

Pooled pharmacokinetic parameters, stratified for children above and below 4 years of age, are presented in Table 3. Young children, as compared to older children, showed a higher body weight-normalized elimination clearance (35 %) and volume of distribution (61 %), with a 14 % lower maximum concentration and a 22 % lower total exposure. Total exposure, normalized on dosage (mg/kg), showed a less pronounced decrease of 15 %. Similar time above MIC was seen while the total exposure above MIC was lower (26 %) in young children compared to older children. However, the previously defined sub-therapeutic plasma concentration threshold of 3 μg/mL [18, 23] was reached in both age groups with an overall exposure in CSF close to that seen in plasma (Fig. 2).

Isoniazid is known to be affected by acetylator status (fast vs slow phenotype) [29–31]. A stratified analysis was therefore performed to evaluate potential pharmacokinetic differences between these two groups composed by 35 fast and 23 slow acetylators in the young group versus 29 fast and 5 slow for older children (Table 4 and Fig. 3). Surprisingly, there was a higher body weight-normalized clearance (20 %) in patients with a slow acetylator phenotype and we observed a 12 % lower dosage-normalized exposure. A large increase of 124 % was also seen for body weight-normalized volume of distribution in patients with a slow acetylator phenotype, resulting in a markedly increased terminal elimination half-life (87 %). However, an imbalance in the number of fast and slow acetylators in the two age groups might have confounded these results. Indeed, the ratio was 60 % fast actetylators in the young age group versus 85 % fast actetylators for the over 4 years old (Table 1). Therapeutic plasma concentrations were reached in both groups and there were no observed differences in the distribution into CSF between fast and slow acetylators.

#### Rifampicin

Overall, rifampicin presented the largest variation in the concentration-time profiles, especially in the younger children (Fig. 4). Pooled pharmacokinetic parameters, stratified for children above and below 4 years of age, are presented in Table 5. Young children, as compared to older children, showed a higher body weight-normalized elimination clearance (25 %) and volume of distribution (29 %), a 18 % lower maximum concentration and a lower total exposure with 16 %. Total exposure, normalized on dosage (mg/kg), showed a less pronounced decrease of only 9 %. Similar times above MIC were observed while the total exposure above MIC was found lower (20 %) in young children compared to older children. Only a few individual concentration measurements and none of the median or interquartile ranges reached the previously defined sub-therapeutic plasma concentration threshold of 8 μg/mL (Fig. 4) [24, 25]. The observed penetration of rifampicin into CSF was very poor with only 2 individual concentration measurements above the wild-type MIC (Fig. 4).

## Discussion

The naïve-pooled pharmacokinetic analysis provided a model-independent analysis approach and was graphically proven to represent the central trends and the variability of the measured concentration-time profiles well in both age-groups and for all drugs. Considerable variability was seen in the observed data which might be explained by the relatively large difference in age and body weight of enrolled children. However, although a naïve pooled data approach provides generally fairly accurate PK estimates [32], the observed variability emphasizes the need to evaluate these treatments on a population level and suggests that a nonlinear mixed-effects approach might provide a more mechanistic understanding of the variability and the influence of clinically important covariates. Pharmacokinetic parameter estimates in this study were generally in agreement with previous studies reporting pharmacokinetic properties of this first-line therapy in children with pulmonary TB [31, 33–35] and TBM [4, 36, 37].

All drugs showed a higher body weight-normalized elimination clearance in young children as compared to older children, and a lower drug exposure was observed in the young group. Similar results have been presented in the literature, noticing a lower exposure in children when compared adults, and lower exposure in young children when compared to older children [30, 31, 38, 39]. These observations lead the revision of the pediatric dosing regimen in TB, recommending an increased dosage for all TB drugs in children. This is most likely a consequence of the well-known allometric relationship between body weight and clearance, and emphasizes the need of dose adjustments in young children with a focus on the youngest patients. In order to produce a dosing alternative that provides an equivalent total drug exposure in children of all age, dosing should therefore rather be scaled with, for example, the body surface area or the lean body weight [40, 41] . Large observed variability and a model-independent analysis, as presented in this study, do not provide a reliable tool to suggest new dosing regimens. However, our data support the recent WHO recommendations to increase the doses of anti-tuberculosis drugs in children with TB, and moreover underline the importance of this in TB meningitis. Further studies are needed urgently to evaluate the clinical impact of the new WHO regimen, designed for pulmonary TB, in young children with TBM.

Plasma concentrations of isoniazid and pyrazinamide reached therapeutic levels rapidly after drug administration. However, only a few individual concentration measurements of rifampicin reached a putative therapeutic level of 8 μg/mL, suggesting that rifampicin may still be severely under-dosed with the current dose recommendation of 15 mg/kg. TBM is primarily a disease of the brain and it is therefore crucial that the administered drugs are distributed from blood into the therapeutic site of action in sufficient concentrations to eliminate the bacterium. Only pyrazinamide and isoniazid demonstrated a large distribution from plasma to CSF with approximately equivalent drug concentrations in plasma and CSF.

Pyrazinamide is only active at low pH which makes definition of MIC difficult in the context of TBM [42]. The role of pyrazinamide within the neutral pH environment of the CSF compartment is unclear. It may have a crucial role in clearing persistent metabolically dormant bacilli within tuberculomas. However, although less frequent in children, pyrazinamide is the antituberculous agent most often implicated in the development of drug-induced liver injuries (DILI) and consideration must therefore be given to the risk of DILI with increased doses of pyrazinamide when determining optimal dosage.

Isoniazid is responsible for killing the majority of the bacteria within the first days of treatment in pulmonary TB [43–46]. Achieving adequate drug concentrations in CSF in the treatment of TBM is therefore likely to be crucial in preventing early mortality and morbidity. This emphasizes the importance of dose-optimization of isoniazid since body weight-normalized clearance and volume of distribution were found significantly higher in younger children, resulting in a decreased exposure. This is consistent with previous reports that younger children eliminate isoniazid faster than older children and adults [30, 47]. Patients with fast acetylator status had a modest decreased body weight-normalized clearance of isoniazid as compared to patients with slow acetylator status (Table 4). These results were unexpected, assuming fast acetylators metabolize isoniazid more rapidly resulting in an increased clearance. However, the results are likely to be confounded by the greater proportion of slow acetylators in the younger age group (40 %) as compared to the older children (15 %). Young children had an overall higher body weight-normalized clearance compared to older children, which may have attenuated the overall difference in isoniazid pharmacokinetics between slow and fast acetylators. Furthermore, Thee et al. previously reported that isoniazid concentrations were not dependent on acetylator status in South African children below 2 years of age [31]. Maturation of the NAT2 enzyme system with age might play an important role in the metabolism of isoniazid and/or there might be ethnic differences in the degree to which NAT2 genotype influences the metabolism of isoniazid. A separate analysis demonstrated an expected 35 % increased body weight-normalized clearance in children with fast acetylator status compared to slow acetylators. It was not deemed reliable to evaluate the fast vs slow phenotype in the older age group considering that only 5 patients of the older children were slow acetylators.

Low concentrations of rifampicin, below the therapeutic threshold, were seen in CSF suggesting higher doses of rifampicin may be more effective in the treatment of childhood TBM. Rifampicin CSF drug concentrations are proportional to dosing and in other forms of TB a dose-related increase in bactericidal activity exists [4, 48] Higher dosages result in increased early bactericidal activity [4, 49] and improved culture conversion rates [50]. A recent Indonesian study showed survival benefit in TBM patients with 13 mg/kg of intravenous rifampicin and additional moxifloxacin for the initial 2 weeks of treatment [7, 51]. These two studies showed the advantage of a higher dosage of rifampicin but here delivered intravenously. An equivalent oral dosage is likely to be between 15 to 25 mg/kg due to the poor and variable bioavailibilty of rifampicin [52, 53]. Children have lower exposure to rifampicin compared to adults, given the same mg/kg dosing. It is likely that dosages of 15–20 mg/kg in children are required to achieve CSF concentration well in excess of the MIC for drug-susceptible strains and higher dosages still (≥20 mg/kg) may be required in very young children [4]. Our findings provide addition support for current and planned studies investigating the use of higher doses for all first-line anti-TB drugs for TBM treatment. The 6-month intensified pediatric regimen used in South Africa with excellent clinical outcomes further supports the hypothesis that higher anti-tuberculosis drug doses, especially of rifampicin, will improve the outcomes of children with TBM [9].

Several limitations of this study deserve comment. First, this was a prospective observational study using an old and no longer recommended regimen. The higher dose regimen recommended by WHO in 2009 was not implemented in Vietnam until 2013. Second, only sparse and small volume CSF sampling was possible and we therefore had to compromise which drugs to analyse. We chose pyrazinamide, isoniazid and rifampicin for a technical reason: ethambutol presents a low molar absorptivity in the UV-visible spectrum and could not be analysed by our LC-UV method. Third, in naïve pooled data analysis, the observations from each individual are grouped or pooled as if it was a single patient. Hence the results from the non-compartmental analysis can only be expressed as group median with no variation. A risk of bias from severe outliers can arise but only with an imbalance of data per individual, which was not the case in this study. Naïve pooled data analysis is a simplistic approach but quickly informative without the need of specialized software and expertise from modelers. However, dedicated population models able to describe the PK/PD of each drug in plasma and CSF of children are on-going. Finally, with few CSF sampling occasions, far from treatment initiation, the relationships between drug exposure, bacterial killing, and clinical outcome are difficult to assess. Most deaths occur rapidly within the first weeks, before any lumbar puncture could be performed giving information on levels of drugs in these patients.

## Conclusion

In summary, this study described the pharmacokinetic properties of isoniazid, pyrazinamide and rifampicin in children with TBM and showed the inadequacy of the relatively low-dose regimens, with an emphasis on the youngest patients below 4 years of age. It is crucial that the administered drugs distribute into the CSF in the treatment of TBM. Only isoniazid and pyrazinamide distributed greatly into the CSF of these enrolled children. This study provided a baseline for evaluation of the pharmacokinetic properties of the new WHO pediatric dosage in Vietnam, which was implemented in 2013. It remains to be confirmed if the new dosing regimen is capable of increasing substantially the drug exposure in the CSF and improving clinical outcomes. More data is urgently needed to establish evidence for optimal drug combinations and doses in the treatment of pediatric TBM.
